# Community-acquired Invasive Bacterial Disease in Urban Gambia, 2005–2015: A Hospital-based Surveillance

**DOI:** 10.1093/cid/ciz463

**Published:** 2019-08-30

**Authors:** Saffiatou Darboe, Uduak Okomo, Abdul-Khalie Muhammad, Buntung Ceesay, Mamadou Jallow, Effua Usuf, Sam Tweed, Edem Akpalu, Brenda Kwambana-Adams, Samuel Kariuki, Martin Antonio, Richard S Bradbury, Karen Forrest, Thushan I de Silva, Bolarinde Joseph Lawal, Davis Nwakanma, Ousman Secka, Anna Roca

**Affiliations:** 1 Medical Research Council Unit The Gambia at the London School of Hygiene and Tropical Medicine, Banjul; 2 Faculty of Infectious and Tropical Diseases, London School of Hygiene and Tropical Medicine, United Kingdom; 3 The School of Medicine, Medical Sciences and Nutrition, University of Aberdeen, United Kingdom; 4 Service de Pediatrie, Centre Hospitalier Universitaire Sylvanus Olypio, Lome, Togo; 5 Kenya Medical Research Institute, Nairobi; 6 School of Medical and Applied Sciences, Central Queensland University, Australia

**Keywords:** invasive bacterial disease, bacteremia, meningitis, community-acquired infection, vaccine preventable disease

## Abstract

***Background.*** Invasive bacterial diseases cause significant disease and death in sub-Saharan Africa. Several are vaccine preventable, although the impact of new vaccines and vaccine policies on disease patterns in these communities is poorly understood owing to limited surveillance data.

***Methods.*** We conducted a hospital-based surveillance of invasive bacterial diseases in The Gambia where blood and cerebrospinal fluid (CSF) samples of hospitalized participants were processed. Three surveillance periods were defined in relation to the introduction of pneumococcal conjugate vaccines (PCVs), before (2005- 2009), during (2010–2011) and after (2012–2015) PCV introduction. We determined the prevalences of commonly isolated bacteria and compared them between the different surveillance periods.

***Results.*** A total of 14 715 blood and 1103 CSF samples were collected over 11 years; overall, 1045 clinically significant organisms were isolated from 957 patients (972 organisms [6.6%] from blood and 73 [6.6%] from CSF). The most common blood culture isolates were *Streptococcus pneumoniae* (24.9%), *Staphylococcus aureus* (22.0%), *Escherichia coli* (10.9%*),* and nontyphoidal *Salmonella* (10.0%). Between the pre-PCV and post-PCV eras, the prevalence of *S. pneumoniae* bacteremia dropped across all age groups (from 32.4% to 16.5%; odds ratio, 0.41; 95% confidence interval, .29–.58) while *S. aureus* increased in prevalence, becoming the most prevalent bacteria (from 16.9% to 27.2%; 1.75; 1.26–2.44). Overall, *S. pneumoniae* (53.4%), *Neisseria meningitidis* (13.7%), and *Haemophilus influenzae* (12.3%) were the predominant isolates from CSF. Antimicrobial resistance to common antibiotics was low.

***Conclusions.*** Our findings demonstrate that surveillance data on the predominant pathogens associated with invasive disease is necessary to inform vaccine priorities and appropriate management of patients.

## Teaser

The etiology of invasive bacterial disease has changed since the introduction of routine pneumococcal vaccines. *Staphylococcus aureus* has emerged as the leading cause of bacteremia.

Invasive bacterial diseases (IBDs) are a leading cause of disease and death, especially among children <5 years of age [1, 2]. Sub-Saharan Africa (SSA) carries a disproportionate burden of these diseases and associated deaths [3]. In addition, microbiological facilities and expertise are scarcely available in many SSA settings [4]. Limited etiological data, necessary to inform prevention strategies, show that several pathogens are associated with IBDs in SSA with diverse distribution profiles across different age groups [2, 5]. The routine use of childhood vaccines, such as *Haemophilus influenzae* type b (Hib) conjugate vaccine and pneumococcal conjugate vaccines (PCVs) [6, 7], has substantially modified the epidemiological profile of owing due to the steep reduction in the prevalence of these 2 bacteria in different age groups [7, 8].

In The Gambia, with the introduction of routine infant immunization with the Hib vaccine in 1997 [9], invasive Hib disease was reduced to negligible levels [10], although a small resurgence was described more than a decade later [11–13]. Subsequently, *Streptococcus pneumoniae, Staphylococcus aureus, E. coli,* and nontyphoidal *Salmonella* (NTS) became the leading causes of bacteremia in the country [14]. In August 2009, a 7-valent PCV was introduced as part of the Expanded Programme on Immunisation (EPI); it was replaced by the 13-valent PCV (PCV13) in 2011. The introduction of PCV13 reduced the incidence of invasive pneumococcal disease by 55% among young children in The Gambia [15]. The impact of PCV introduction in The Gambia on bacterial diseases in older children and adults, owing to the vaccine’s herd effect, has not yet been described.

The increasing threat from antimicrobial resistance remains a global challenge, resulting in longer durations of illness, mortality, and prophylaxis failure [16]. This is particularly so in SSA, where the burden is substantial, the choice of effective antimicrobials is limited, and surveillance data for invasive infections are lacking [17–19]. In addition to data on the prevalent bacterial pathogens associated with community and hospital-acquired invasive disease, hospital-based surveillance in resource limited settings should therefore include resistance patterns to commonly used antibiotics.

This study provides data on the main causes of postneonatal IBD in The Gambia and antibiotic susceptibility patterns over an 11-year period, between 2005 and 2015, during which PCV was introduced in the country. The data presented here are part of ongoing facility-based IBD surveillance.

## METHODS AND MATERIALS

### Study Setting and Population

The Gambia is a subtropical country in West Africa with a single wet season from June to October. Malaria is endemic, and peak transmission occurs from July to November during the rains; however, because of scaling up of malaria control interventions, a substantial decline has been observed in recent years [20]. Malnutrition remains a problem, with the prevalence of underweight, stunting, and wasting among children <5 years old estimated at 16.4%, 25.0%, and 4.3%, respectively [21]. The prevalence of human immunodeficiency virus (HIV) among adults aged 15–49 years remains low and was estimated at 2.1% in 2015 [22].Vertical transmission is low among mothers with HIV receiving prophylaxis and treatment [23]. Infant EPI vaccine coverage is high, above 95% for the BCG vaccine and above 90% and 80%, respectively, for a single dose and 3 doses of the diphtheria-pertussis-tetanus vaccine in all regions [24]. The Hib vaccine is given at birth and at 2, 3, 4, and 16 months of age, and the PCV at 2, 3, and 4 months of age. The vaccine against *Neisseria meningitidis* group A (MenAfriVac) has been used in mass campaigns during outbreaks but has not been rolled out as part of the EPI schedule [10, 25, 26].

This hospital-based surveillance was conducted at the Clinical Services Department (CSD) of the Medical Research Council (MRC) Unit The Gambia (MRCG) at the London School of Hygiene and Tropical Medicine, situated 12 km from the capital, Banjul. The CSD has provided primary- and secondary-level care to sick individuals from the surrounding population, MRCG research study participants, ad a small number of patients referred from other clinics since the late 1950s. Approximately 50 000 patients of all ages are seen each year in the outpatient department, and 1400 are hospitalized in the 42-bed ward. It is the only health facility in The Gambia where microbiological cultures are routinely obtained in patients with suspected IBD. Blood and cerebrospinal fluid (CSF) samples are routinely collected for bacterial culture from patients with suspected sepsis and meningitis. 

Patients with suspected sepsis are treated empirically with ampicillin and gentamicin, and those with suspected meningitis are treated empirically with ceftriaxone. Treatment is subsequently modified by clinical response and laboratory results. MRCG research study participants are recruited from other surrounding health facilities, with clinical samples sent to the MRCG clinical laboratories for processing using similar methods to those used for non–research study patients. Blood and CSF samples are collected only from referred patients who require admission on the MRC ward.

### Microbiological Procedures

As part of this surveillance, bacterial isolates were obtained from blood using an automated blood-culture system (BACTEC 9050; Becton Dickinson), following the manufacturer’s instructions for quality control and blood volume requirements. Commercially produced BD BACTEC PEDS/Plus/F culture bottles were used for specimens obtained from children (aged 1 month to 15 years) and BD BACTEC Plus Aerobic/F* and Plus Anaerobic/F* culture bottles for specimens from adults (aged >15 years), as described elsewhere [14, 27]. CSF samples were processed according to World Health Organization protocol [28]. Standard microbiological procedures were performed, as described elsewhere for all pathogens [10]. In summary, pneumococcal isolates were identified using optochin disk susceptibility tests on blood agar in 5% carbon dioxide and bile solubility tests to confirm resistant isolates [27]*. H. influenzae* were serotyped by latex agglutination [10], and *S. aureus* were identified using coagulase and mannitol. For other isolates, further identification was done as appropriate for the pathogen. All normal skin flora isolates (coagulase-negative staphylococci, *Bacillus* species, *Micrococcus* spp., diphtheroids, *Propionibacterium* spp., and *Bacillus* spp. other than *Bacillus anthracis*) were regarded as clinically nonsignificant. 

Antimicrobial sensitivity patterns were determined by means of Kirby-Bauer disk diffusion on Mueller-Hinton agar and interpreted according to the relevant Clinical Laboratory Standard Institute guidelines on antimicrobial agents [29]. Antibiotics tested as relevant to each pathogen included ampicillin, gentamicin, tetracycline, cotrimoxazole, chloramphenicol, ciprofloxacin, cefoxitin (to infer susceptibility to methicillin), and cefotaxime (BD Oxoid). Appropriate American Type Culture Collection controls were consistently used for the antibiotic susceptibility testing. Invasive bacteria isolates (blood and CSF) were stored at −70°C as part of routine microbiological surveillance. Samples were processed at the clinical microbiology laboratory, which is Good Clinical Laboratory Practice (2010) and ISO (International Organization for Standardization) 15189 (2015) accredited and submits to the external quality control assessment of the Kenya Accreditation Service in accordance with international quality systems for laboratories.

### Statistical Analysis

Data were extracted from clinic and laboratory databases for research study participants and non–research study patients. All relevant invasive bacterial isolates within the study period were included in the analysis. We assumed each presentation of a patient as independent but considered patients with multiple positive cultures with the same pathogen, obtained within 4 weeks of each other, as the same episode, which was therefore only reported once. We also considered an observation to be repeated if the same patient was associated with different specimen types (blood and CSF sample). 

Bacterial etiology patterns and trends were compared between surveillance periods defined by the introduction of PCVs, as follows: before PCV introduction (January 2005 to December 2009), PCV introduction and rollout (January 2010 to December 2011), and after PCV introduction (January 2012 to December 2015). Crude odds ratios for IBD, comparing post-PCV and pre-PCV periods, were obtained using logistic regression. To address confounding by age, we stratified the analyses by age. Missing values were excluded likewise, and because <1% of variables had missing values, imputation methods were not considered, and a complete case analysis was deemed adequate. The descriptive analysis for this study were carried out using Stata 13.1 software and the R Version 3.4.4 Plotly package for the graphs.

### Ethical Review and Approval

Clinical samples were collected for standard clinical management, and the results were anonymized for analytical purpose. The surveillance received ethical approval from the joint MRC/Gambia government ethics committee.

## RESULTS

**Figure 1.**
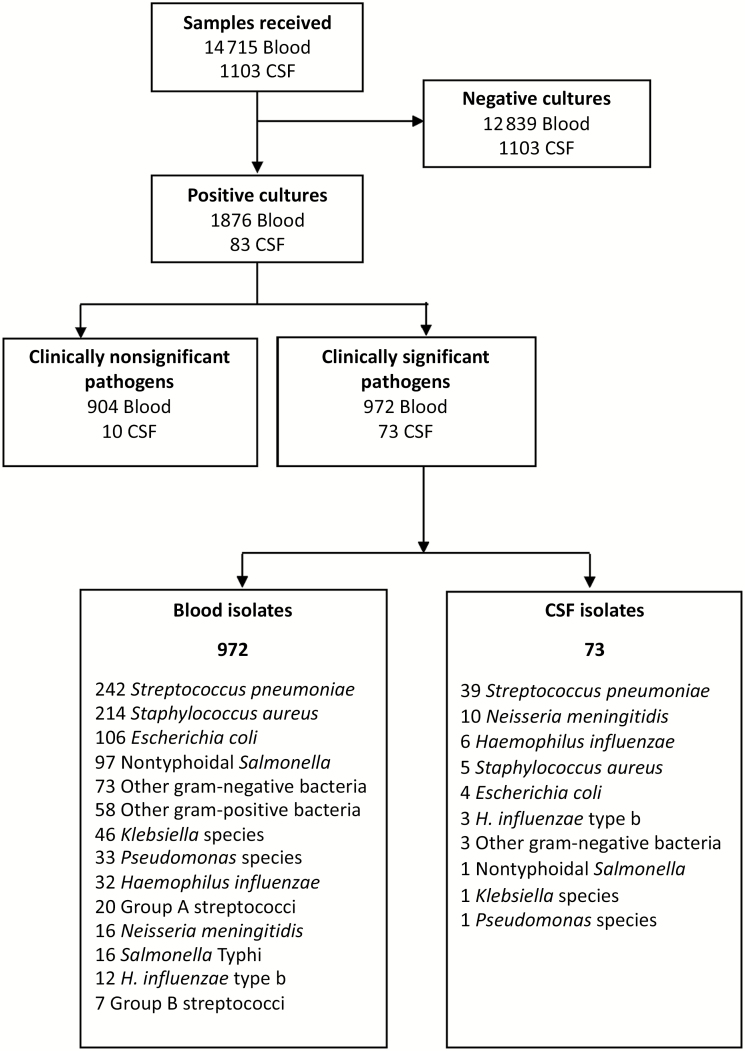
Sampling profile and pathogen outcome from blood and cerebrospinal fluid (CSF).

Between January 2005 and December 2015, a total of 14 715 blood and 1103 CSF samples were processed for bacterial culture (Figure 1). The median age of patients with IBD was 4 years (interquartile range, 1–31 years).

### Bacteremia

**Figure 2.**
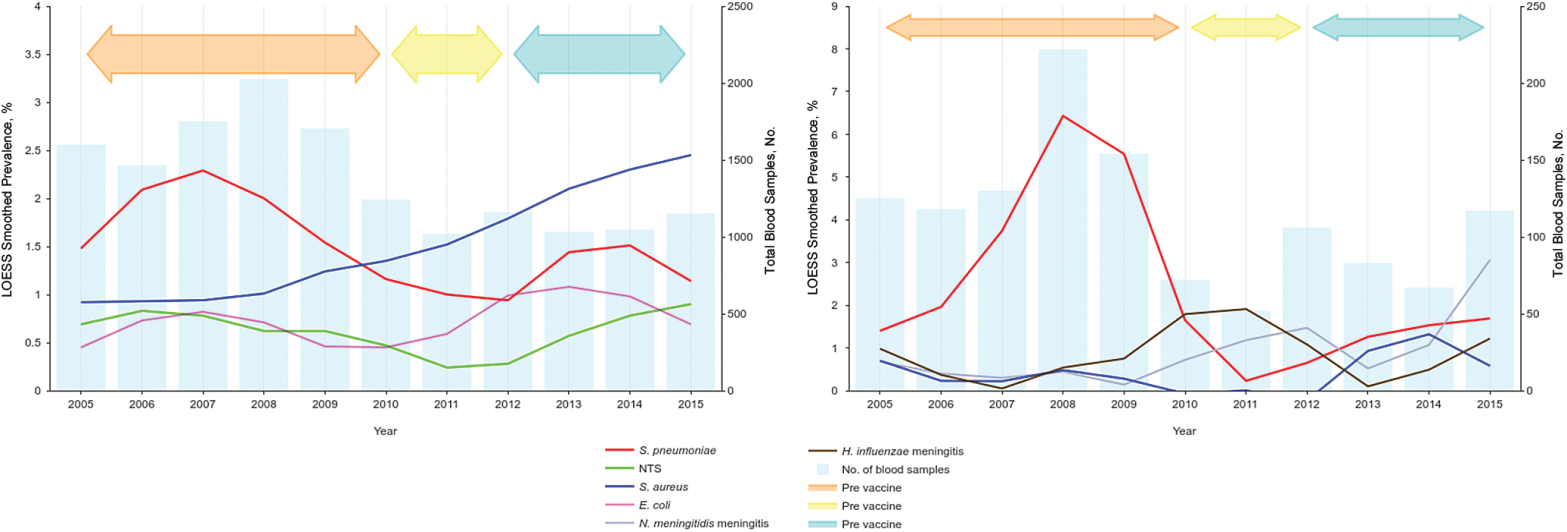
Annual trends in major bacterial pathogens associated with invasive bacterial infections in The Gambia, 2005–2015, in relation to the introduction of pneumococcal conjugate vaccines. *A*, Bacteremia. *B*, Meningitis.

The number of blood culture samples processed was higher during the pre-PCV period (2005–2009) than during the post-PCV period, peaking in 2008 (Figure 2A). Of the samples cultured, 1876 (12.7%) were positive for any pathogen, of which 972 (51.8%) were considered clinically significant and 904 (48.2%) clinically nonsignificant or contaminants. The overall prevalence of clinically significant bacteremia was 6.6% (972 of 14 715) and ranged from 5.9% in the pre-PCV to 8.5% in the post-PCV period. The predominant clinically significant bacterial isolates in blood cultures were *S. pneumoniae* (25% [242 of 972]), *S. aureus* (22% [214 of 972]), *E. coli* (11% [106 of 972]), and NTS (10% [97 of 972]) (Figure 1). We also observed a seasonal pattern of infections, with *S*. *pneumoniae* more common during the dry season and *S. aureus* infections more common during the wet season (Supplementary Figure 1). However, exceptions were observed in 2010 and 2011 for *S. aureus,* where the overlap started, and in 2014 for *S. pneumoniae,* which saw a steep surge in prevalence (Figure 2A). No distinct seasonal pattern was observed with other organisms.

Overall and age-specific prevalences of all blood culture isolates by vaccine surveillance period re summarized in Table 1. In addition to the significantly decreased odds of *S. pneumoniae* bacteremia (odds ratio, 0.41; 95% confidence interval, .29–.58) and increased odds of *S. aureus* bacteremia (1.75; 1.26–2.44) among all age groups in the post-PCV surveillance period, we observed a 9-fold increase in the odds of bacteremia due to *N. meningitidis*. We observed no change in the *E. coli* but did note a decrease in NTS. When results were stratified by age, the most significant reduction in *S. pneumoniae* bacteremia was noted among children aged 2–23 months and adults ≥15 years. Likewise, the increases in *S*. *aureus* bacteremia was among children aged 2–23 months. No differences were observed between study periods for other common bacteria. The prevalence of the predominant pathogens by year is summarized in Figure 2A and shows a sustained increase on *S. aureus* bacteremia over the study periods along with a decline in *S. pneumoniae*.

**Table 1.** Distribution of Blood Pathogens in the Different Vaccine Periods by Age Group

Pathogen by Age Group	Pathogens, No. (%)				OR for Post-PCV vs Pre-PCV Periods	95% CI	Change in Post-PCV vs Pre-PCV Periods, %	*P* Value
	Total.	Pre-PCV Period	PCV Introduction	Post-PCV Period				
All age groups	972	494	126	352	…	…	…	…
* Streptococcus pneumoniae*	242	160 (32.4)	24 (19.0)	58 (16.5)	0.41	.29–.58	−15.9	<.001
* Staphylococcus aureus*	214	87 (16.9)	31 (23.4)	96 (27.2)	1.75	1.26–2.44	10.3	<.001
* Escherichia coli*	106	55 (11.1)	12 (9.5)	39 (11.1)	0.99	.64–1.54	0.0	.98
* *NTS	97	61 (12.3)	8 (6.3)	28 (8.0)	0.61	.38–.98	−4.3	.04
* *Other gram-negative bacteria	73	30 (6.1)	7 (5.6)	36 (10.2)	1.76	1.06–2.92	4.1	.03
* *Other gram-positive bacteria	58	14 (2.8)	18 (14.3)	26 (7.4)	2.73	1.41–5.32	4.6	.002
* Klebsiella* spp.	46	20 (4.0)	5 (4.0)	21 (6.0)	1.50	.80–2.82	2.0	.20
* Pseudomonas* spp.	33	25 (5.1)	4 (3.2)	4 (1.1)	0.22	.07–.63	−4.0	<.001
* Haemophilus influenzae* non– type b	32	9 (1.8)	8 (6.3)	15 (4.3)	2.40	1.04–5.55	2.5	.04
* *GAS	20	14 (2.8)	1 (0.8)	5 (1.4)	0.49	.18–1.38	−1.4	.16
* Neisseria meningitidis*	16	2 (0.4)	1 (0.8)	13 (3.7)	9.43	2.12– 42.07	3.3	<.001
* Salmonella* Typhi	16	9 (1.8)	4 (3.2)	3 (0.9)	0.46	.12–1.72	−0.9	.23
* H. influenzae* type b	12	5 (1.0)	1 (0.8)	6 (1.7)	1.70	.51–5.60	0.7	.38
* *GBS	7	3 (0.6)	2 (1.6)	2 (0.6)	0.94	.16–5.63	0.0	.94
2–23 mo	332	173	39	120	…	…	…	…
* S. aureus*	92	38 (22.0)	11 (28.2)	43 (35.8)	1.98	1.18–3.33	13.8	.009
* S. pneumoniae*	81	57 (32.9)	7 (17.9)	17 (14.2)	0.34	.18–.61	−18.7	<.001
* *NTS	26	15 (8.7)	4 (10.3)	7 (5.8)	0.65	.26–1.65	−2.9	.36
* E. coli*	26	14 (8.1)	1 (2.6)	11 (9.2)	1.15	.50–2.62	1.1	.75
* H. influenzae* non–type b	21	5 (2.9)	7 (17.9)	9 (7.5)	2.72	.89–8.34	4.6	.07
* *Other gram-negative bacteria	19	5 (2.9)	2 (5.1)	12 (10.0)	3.73	1.28–10.89	7.1	.01
* *Other gram-positive bacteria	17	10 (5.8)	1 (2.6)	6 (5.0)	0.86	.30–2.43	−0.8	.77
* Klebsiella* spp.	16	8 (4.6)	2 (5.1)	6 (5.0)	1.09	.37–3.21	0.4	.88
* Pseudomonas* spp.	10	8 (4.6)	1 (2.6)	1 (0.8)	0.17	.02–1.40	−3.8	.12
* *GAS	9	7 (4.0)	0 (0)	2 (1.7)	0.40	.08–1.97	−2.3	.23
* H. influenzae* type b	6	3 (1.7)	1 (2.6)	2 (1.7)	0.96	.16–5.84	0.0	.96
* *GBS	5	2 (1.2)	1 (2.6)	2 (1.7)	1.45	.20–10.43	0.5	.71
* N. meningitidis*	3	1 (0.6)	0	2 (1.7)	1.06	.26–32.52	1.1	.37
* S.* Typhi	1	0	1 (2.6)	0	…	…	NA	…
24–59 mo	145	61	27	57	…	…	…	…
* S. pneumoniae*	41	20 (32.8)	7 (25.9)	14 (24.6)	0.67	.30–1.49	−8.2	.32
* S. aureus*	33	10 (16.4)	9 (33.3)	14 (24.6)	1.66	.67–4.11	8.2	.27
* *NTS	18	10 (16.4)	1 (3.7)	7 (12.3)	0.71	.25–2.02	−4.1	.52
* *Other gram-positive bacteria	10	2 (3.3)	4 (14.8)	4 (7.0)	2.23	.39–12.65	3.7	.35
* E. coli*	8	2 (3.3)	2 (7.4)	4 (7.0)	2.23	.39–12.65	3.7	.35
* *Other gram-negative bacteria	8	6 (9.8)	1 (3.7)	1 (1.8)	0.16	.02–1.40	−8.0	.05
* N. meningitidis*	7	1 (1.6)	1 (3.7)	5 (8.8)	5.77	.64–50.98	7.2	.07
* Klebsiella* spp.	6	2 (3.3)	1 (3.7)	3 (5.3)	1.64	.26–10.19	2.0	.59
* H. influenzae* non–type b	4	3 (4.9)	0	1 (1.8)	0.35	.03–3.42	−3.1	.33
* *GAS	4	1 (1.6)	1 (3.7)	2 (3.5)	2.18	.19–24.74	1.9	.52
* Pseudomonas* spp.	2	1 (1.6)	0	1 (1.8)	1.07	.07–17.54	0.2	.96
* H. influenzae* type b	2	1 (1.6)	0	1 (1.8)	1.07	.07–17.54	0.2	.96
* *GBS	1	1 (1.6)	0	0	1.00	1.00–1.00	NA	…
* S*. Typhi	1	1 (1.6)	0	0	1.00	1.00–1.00	NA	…
5–14 y	127	45	23	59	…	…	…	…
* S. aureus*	42	13 (28.9)	6 (26.1)	23 (39.0)	1.57	.69–3.61	10.1	.28
* S. pneumoniae*	24	13 (28.9)	3 (13.0)	8 (13.6)	0.39	.14–1.03	−15.3	.054
* *Other gram-negative bacteria	14	5 (11.1)	3 (13.0)	6 (10.2)	0.91	.26–3.18	−0.9	.88
* *NTS	11	6 (13.3)	2 (8.7)	3 (5.1)	0.35	.08–1.48	−8.2	.14
* *Other gram-positive bacteria	9	0	5 (21.7)	4 (6.8)	1.00	1.00–1.00	NA	NA
* S.* Typhi	6	3 (6.7)	2 (8.7)	1 (1.7)	0.24	.02–2.40	−5.0	.19
* E. coli*	5	0	1 (4.3)	4 (6.8)	1.00	1.00–1.00	NA	*…*
* Klebsiella* spp.	4	2 (4.4)	0	2 (3.4)	0.75	.10–5.57	−1.0	.78
* Pseudomonas* spp.	4	2 (4.4)	1 (4.3)	1 (1.7)	0.37	.03–4.22	−2.7	.42
* N. meningitidis*	4	0	0	4 (6.8)	1.00	1.00–1.00	NA	*…*
* H. influenzae* non–type b	3	0	0	3 (5.1)	1.00	1.00–1.00	NA	*…*
* H. influenzae* type b	1	1 (2.2)	0	0	1.00	1.00–1.00	NA	*…*
* *GAS	0	0	0	0	NA	NA	NA	NA
* *GBS	0	0	0	0	NA	NA	NA	NA
≥15 y	359	210	36	113	…	…	…	…
* S. pneumoniae*	93	69 (32.9)	6 (16.7)	18 (15.9)	0.39	.22–.69	−17.0	<.001
* E. coli*	66	38 (18.1)	8 (22.2)	20 (17.7)	0.97	.54–1.77	−0.4	.93
* S. aureus*	47	26 (12.4)	5 (13.9)	16 (14.2)	1.17	.60–2.28	1.8	.65
* *NTS	42	30 (14.3)	1 (2.8)	11 (9.7)	0.65	.31–1.35	−4.6	.23
* *Other gram-negative bacteria	30	12 (5.7)	1 (2.8)	17 (15.0)	2.92	1.34–6.36	9.3	.006
* *Other gram-positive bacteria	22	2 (1.0)	8 (22.2)	12 (10.6)	10.94	2.38– 50.33	9.6	<.001
* Klebsiella* spp.	18	7 (3.3)	2 (5.6)	9 (8.0)	2.51	.91–6.93	4.7	.08
* Pseudomonas* spp.	17	14 (6.7)	2 (5.6)	1 (0.9)	0.12	.02–.96	−5.8	.008
* S.* Typhi	8	5 (2.4)	1 (2.8)	2 (1.8)	0.74	.14–3.87	−0.6	.72
* *GAS	7	6 (2.9)	0	1 (0.9)	0.30	.04–2.55	−2.0	.21
* H. influenzae* non–type b	3	1 (0.5)	1 (2.8)	1 (0.9)	1.87	.12–.12	0.4	.66
* H. influenzae* type b	3	0	0	3 (2.7)	1.00	1.00–1.00	NA	…
* N. meningitidis*	2	0	0	2 (1.8)	1.00	1.00–1.00	NA	…
* *GBS	1	0	1 (2.8)	0	…	…	NA	…

Abbreviations: CI, confidence interval; GAS, group A streptococci; GBS, group B streptococci; NA, not available; NTS, nontyphoidal *Salmonella*; OR, odds ratio; PCV, pneumococcal conjugate vaccine.

### Meningitis

Eighty-three (7.5%) of CSF samples cultured were positive, the majority (87.9% [73 of 83]) of which were considered clinically significant, representing 6.6% of the overall samples. Overall, the most common clinically significant isolates were *S. pneumoniae* (53% [39 of 73]), *N. meningitidis* (14% [10 of 73]), *and H. influenzae* (12% [9 of 73]) (Table 2). We observed a considerably higher number of CSF samples processed in the pre-PCV period, during which there was a corresponding increase in the isolation of *S. pneumoniae* (Figure 2B). *S. pneumoniae* was the predominant cause of meningitis in the pre-PCV period (67% [33 of 49] compared with 26% [5 of 19] after PCV introduction; *P* = .002). However, the prevalence of *S. pneumoniae* meningitis gradually declined from 2008, before the introduction of PCV. *N. meningitidis* increased in the post-PCV compared with the pre-PCV period (from 6.1% to 31.6%; odds ratio, 7.08; 95% confidence interval, 1.55–32.24), becoming the most prevalent bacteria in this latter period.

**Table 2.** Distribution of Cerebrospinal Fluid Pathogens in the Different Vaccine Periods (All Age Groups)

Pathogen	Pathogens, No. (%)				OR for Post-PCV vs Pre-PCV Periods	95% CI	Change in Post-PCV vs Pre-PCV Periods, %	*P* Value
	Total	Pre-PCV Period	PCV Introduction	Post-PCV Period				
All pathogens	73	49	5	19	…	…	…	…
*Streptococcus pneumoniae*	39	33 (67.3)	1 (20.0)	5 (26.3)	0.17	.05–.57	−41.0	.002
*Neisseria meningitidis*	10	3 (6.1)	1 (20.0)	6 (31.6)	7.08	1.55–32.24	25.5	.009
*Haemophilus influenzae* non– type b	6	3 (6.1)	2 (40.0)	1 (5.3)	0.85	.85–8.74	−0.8	.89
*Staphylococcus aureus*	5	3 (6.1)	0	2 (10.5)	1.80	.28–11.75	4.4	.55
*Escherichia coli*	4	2 (4.1)	1 (20.0)	1 (5.3)	1.31	.11–15.30	1.2	.83
*H. influenzae* type b	3	2 (4.1)	0 (0.0)	1 (5.3)	1.31	.11–15.30	1.2	.83
Other gram-negative bacteria	3	1 (2.0)	0	2 (10.5)	5.65	.48–66.32	8.5	.55
Nontyphoidal *Salmonella*	1	1 (2.0)	0	0	1.00	1.00–1.00	NA	NA
*Klebsiella* spp.	1	0	0	1 (5.3)	1.00	1.00–1.00	NA	NA
*Pseudomonas* spp.	1	1 (2.0)	0	0	1.00	1.00–1.00	NA	NA

Abbreviations: CI, confidence interval; NA, not available; OR, odds ratio; PCV, pneumococcal conjugate vaccine.

### Dual Infections and Coinfections

The same pathogen was isolated from concomitant blood and CSF samples of 36 patients (17 S*. pneumoniae*, 6 *N. meningitidis*, 5 *H. influenzae*, and 4 *S. aureus*). Twenty-three patients had coinfections with >1 pathogen; *S. aureus* was the most common (n = 12), coinfected with group A or group B streptococci (each n = 3), *S. pneumoniae* (n = 3), and NTS, *H. influenzae,* and *N. meningitides* (each n = 1).

### Antimicrobial Resistance

**Figure 3.**
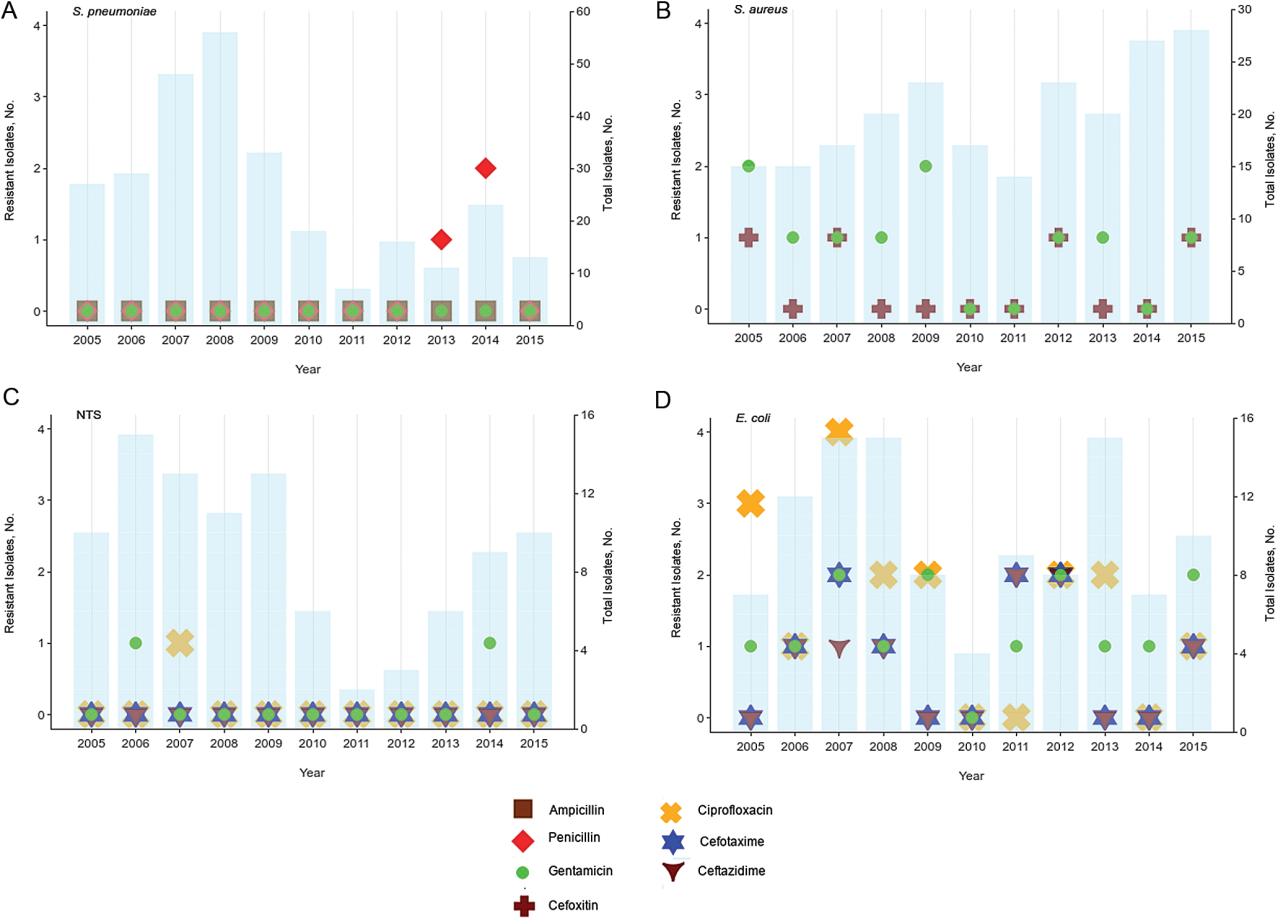
Antibiotic resistance patterns of the major causes of invasive bacterial infection in The Gambia, 2005–2015. *A*, *Streptococcus pneumoniae*. *B*, *Staphylococcus aureus*. *C*, Nontyphoidal *Salmonella* (NTS). *D*, *Escherichia coli*.

Antimicrobial resistance was low for the clinically relevant antimicrobial. as recommended by the World Health Organization [30]. *S. pneumoniae* was highly sensitive to penicillin, ampicillin, and gentamicin (Figure 3A). Similarly, *S. aureus* was sensitive to cefoxitin, the surrogate for methicillin and gentamicin (Figure 3B). Only 4 cases of invasive methicillin-resistant *S. aureus* (1.9%) were found. In addition, resistance to gentamicin and cephalosporins was low for NTS (Figure 3C) and *E. coli* (Figure 3D). There was no increase in resistance over the study periods.

## DISCUSSION

This study provides 11 years of data on the trends of the major clinically significant pathogens responsible for IBD among inpatients in urban Gambia. For the first time we have shown how these major bacteria have changed in relation to the introduction of PCV, with *S. aureus and N. meningitidis* replacing *S. pneumoniae* as the major causes of bacteremia and meningitis, respectively. The findings of the current study underscore the need for routine hospital microbiological surveillance.

The decline of *S. pneumoniae* as the main cause of bacteremia mirrors the corresponding rise in the predominance of *S. aureus*. Population-based surveillance in rural Gambia has shown a 55% decrease in the incidence of invasive pneumococcal disease among infants aged 2–23 months after the introduction of PCV [15]. Our hospital-based surveillance data support the previous denominator-based findings and, in addition, show a decrease in the prevalence of invasive disease among nonvaccinated older children and adults. 

Interestingly, the decrease in *S. pneumoniae* prevalence, along with the increase in *S. aureus,* started before the introduction of PCVs. The reason for these changes remain unclear, given that no major public health interventions that could have resulted in a rapid epidemiological shift were introduced around the same time. Nevertheless, years after the introduction of the wider serotype-covering PCV13, *S. pneumoniae* remains an important cause of invasive disease across all age groups (second most cause of both bacteremia and meningitis). Recent data from The Gambia have shown persistence of nasopharyngeal carriage of pneumococci of vaccine serotypes along with an increased carriage of nonvaccine serotypes [31]. Because carriage is a prerequisite for invasive disease, those data, along with our results, suggest that further improvement to widen the coverage of serotypes and schedule of PCV could have an additional impact.

*S. aureus* was prevalent over the surveillance period but emerged as the primary cause of bacteremia in the post-PCV era being isolated in 30% of proven bacteremia cases among infants 2–23 months old. Although our study design precludes documenting an increase incidence of *S. aureus* bacteremia, there is a global increase in incidence with a changing epidemiology attributed to a range of factors such as vaccines, virulence, invasive procedures, antibiotic resistance, and immune suppression [32, 33], underlying an urgent need for further studies and improved preventive strategies. In particular, the underlying source of *S. aureus* bacteremia cases in low- and middle-income countries warrants further investigation, because these may be distinct from the prevalent causes in high-income countries, such as intravenous drug use and nosocomial infections related to intravenous devices and catheters. Other factors, such as skin and soft-tissue infections, bone and joint infections, and endocarditis [34], are implicated in methicillin-susceptible *S. aureus* bacteremia.

*E. coli* and NTS were also important causes of bacteremia in our setting. Although the prevalence of *E. coli* has remained stable, that of NTS significantly declined after PCV introduction, with the overall number of cases relatively small (n = 97). This is consistent with previous reports from rural Gambia [35], in which it has been associated with the decline in malaria infection, because malaria transmission is relatively low in the study setting [20]. In addition, multiple host risk factors described for invasive NTS, such as HIV infection and poor sanitation, are low in prevalence, and with documented improvement in the latter [22, 36]. Only 10 cases of meningitis due to *N. meningitidis* were observed over the entire surveillance period, and these coincided with an outbreak in the Eastern region. More than 2 decades after the Hib vaccine was introduced, with near-elimination of invasive disease in The Gambia and neighboring Senegal [10, 37], sporadic cases still occur, reinforcing the need for continued surveillance.

Antimicrobial resistance was generally low, including very few methicillin-resistant *S. aureus* isolates compared with some SSA countries [17, 38]. Antimicrobial resistance for NTS in our setting has been described elsewhere [39], and resistance to third-generation cephalosporins was low for *E. coli*. However, overall ciprofloxacin resistance for *E. coli* may warrant monitoring resistance quinolones for probable emergence of multidrug resistance [40]. Although resistance did not significantly increase over the study period, overprescription of antibiotics needs to be monitored, and stringent control measures should be in place to encourage the use of guidelines [41], because alternative antibiotics strategies are limited in resource-limited countries such as The Gambia.

The current study had several limitations intrinsic to the surveillance design. Because our data are hospital based, the observed changes in pathogen prevalence were dependent on sampling and may not reflect changes in disease incidence. It is a retrospective analysis, and any changes in case ascertainment, amount of sample volume collected, or health-seeking behavior in the population may have modified the overall yield of bacteria. Although the case ascertainment for patients seen at the MRCG CSD did not change over the surveillance period, there were several research studies during the period, each with different age inclusion criteria. Although this might not have changed the distribution of pathogens, it probably increased the number of isolates in some years compared with others. The linking of clinical data associated with microbiological findings is an another limitation. In addition, we could not determine what proportion of patients had received the PCV. Finally, we determined antimicrobial sensitivity using disk diffusion without final confirmation by Etest, which may have resulted in an overestimation of the prevalence of antibiotic resistance levels. Still, results show a low prevalence of antibiotic resistance and no trends for an increase.

Our long surveillance data have shown that *S. aureus* and *N. meningitis* have emerged as the leading causes of bacteremia and meningitis, respectively, in urban Gambia, while *S. pneumoniae* remains a leading cause of IBD, even after the introduction of PCV13. The changing epidemiology of IBD makes a compelling case for regular microbiological and antimicrobial surveillance data, which is lacking in SSA. Not only are such data necessary for healthcare workers to inform appropriate antibiotic prescribing practices, but they are also vital for prioritizing vaccine development, for emerging pathogens such as *S. aureus* and optimizing schedules of current vaccines. New vaccines targeting *S. aureus* should focus on young infants and older children, among whom the prevalence of IBD is highest.

## Supplementary Data

Supplementary materials are available at *Clinical Infectious Diseases* online. Consisting of data provided by the authors to benefit the reader, the posted materials are not copyedited and are the sole responsibility of the authors, so questions or comments should be addressed to the corresponding author.

ciz463_suppl_Supplemental-Figure-1

## Notes

***Author contributions.*** S. D., U. O., R. S. B., O. S., and A. R. designed the study and wrote the manuscript. S. D. and U. O. prepared the tables and figures, S. D. and A. K. M. analyzed the data. S. D., B. C., M. J., and O. S. performed the laboratory analysis. All authors reviewed and contributed to the manuscript. R. S. B. coauthored this manuscript in his personal capacity and in his role as an adjunct academic at Central Queensland University.

***Acknowledgments.*** The authors thank Ameh James for his comments and suggestions, Kabiro Kanteh and Fatoumata Ceesay for helping retrieve records, and the staff of the Clinical Services Department and Clinical Laboratories.

***Financial support.*** This work was supported by the Medical Research Council Unit The Gambia at the London School of Hygiene and Tropical Medicine.

***Supplement sponsorship.*** This supplement was supported with funds from Gavi, the Vaccine Alliance through The World Health Organization and the CDC Foundation, and The Medical Research Council Unit The Gambia at the London School of Hygiene and Tropical Medicine.

***Potential conflicts of interest.*** All authors: No reported conflicts. All authors have submitted the ICMJE Form for Disclosure of Potential Conflicts of Interest. Conflicts that the editors consider relevant to the content of the manuscript have been disclosed.
