# Supplementary figures and images for: Community-acquired Invasive Bacterial Disease in Urban Gambia, 2005–2015: A Hospital-based Surveillance
Source: Clin Infect Dis. 2019 Aug 30;69(Suppl 2):S105–13. doi: 10.1093/cid/ciz463 (PMC6761311; doi:10.1093/cid/ciz463)

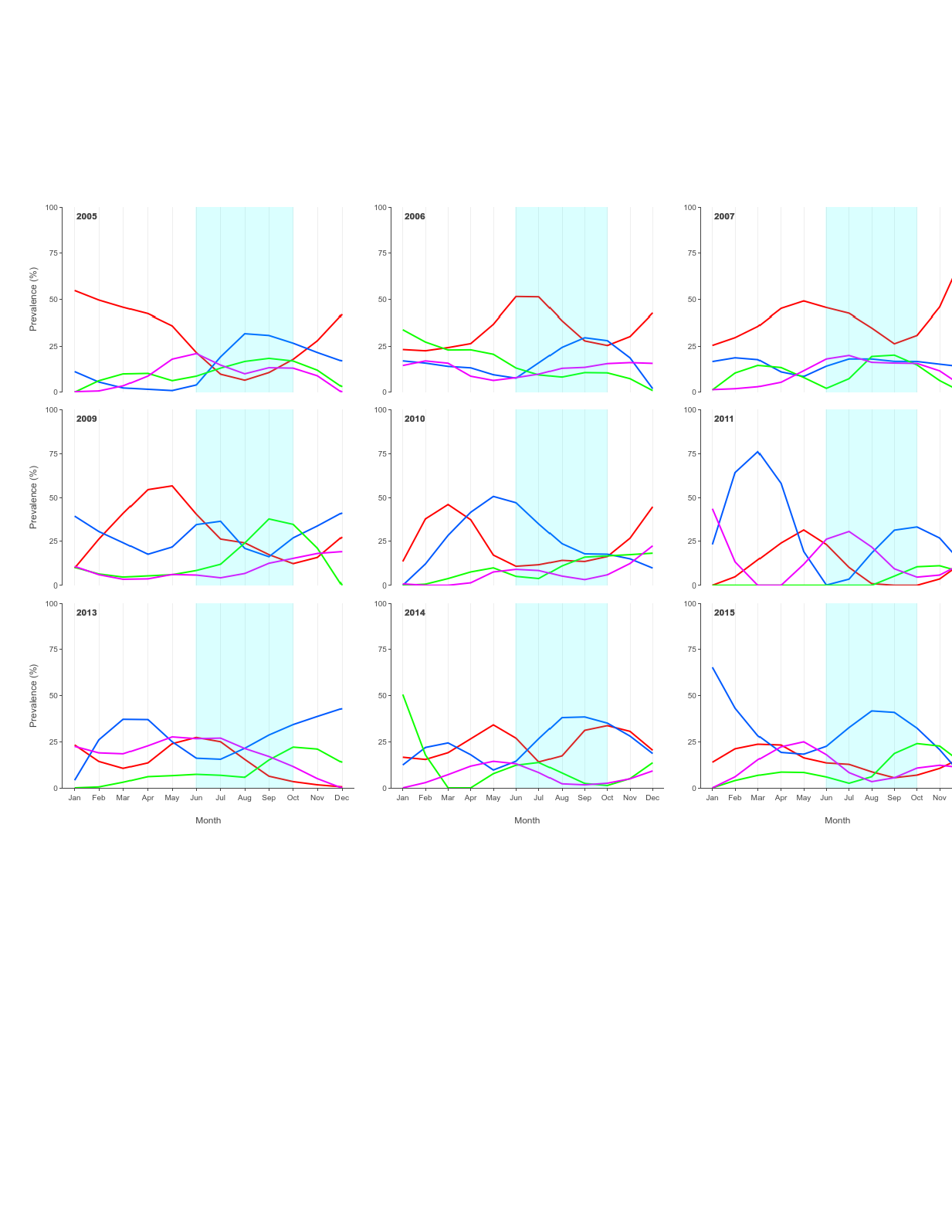

Supplement: ciz463_suppl_Supplemental-Figure-1 [file ciz463_suppl_supplemental-figure-1.png]
